# CMV-RNAemia as new marker of active viral replication in transplant recipients

**DOI:** 10.1128/jcm.01630-23

**Published:** 2024-03-27

**Authors:** Giulia Piccirilli, Federica Lanna, Liliana Gabrielli, Vincenzo Motta, Martina Franceschiello, Alessia Cantiani, Matteo Pavoni, Marta Leone, Eva Caterina Borgatti, Dino Gibertoni, Renato Pascale, Maddalena Giannella, Francesca Bonifazi, Tiziana Lazzarotto

**Affiliations:** 1 Microbiology Unit, IRCCS Azienda Ospedaliero-Universitaria di Bologna, Bologna, Italy; 2 Department of Medical and Surgical Sciences, Alma Mater Studiorum, University of Bologna, Bologna, Italy; 3 Research and Innovation Unit, IRCCS Azienda Ospedaliero-Universitaria di Bologna, Bologna, Italy; 4 Infectious Diseases Unit, Department for Integrated Infectious Risk Management, IRCCS Azienda Ospedaliero-Universitaria di Bologna, Bologna, Italy; 5 IRCCS Azienda Ospedaliero-Universitaria di Bologna, Istituto di Ematologia “Seràgnoli”, Bologna, Italy; Mayo Clinic Minnesota, Rochester, Minnesota, USA

**Keywords:** cytomegalovirus, CMV-RNAemia, CMV-DNAemia, transplant, letermovir

## LETTER

Currently, the quantification of cytomegalovirus (CMV) DNA in blood samples (CMV-DNAemia) represents the gold standard for identifying active viral replication, preventing CMV-related disease, and monitoring response to drugs targeting CMV-DNA polymerase (1–5). The recent introduction of letermovir (LMV) showed that CMV-DNAemia may not be an accurate marker of active viral replication (6–9). Indeed, by blocking the terminase complex, LMV induces the release of free CMV-DNA fragments (abortive infection), which could lead to potential misinterpretation of molecular testing results (1, 6–9). Additional methods, such as CMV-viremia (shell vial method) and CMV-DNAemia post-DNase (DNase test), could be used to prove active viral replication. CMV viremia detects CMV infectious particles in cell culture, whereas the DNase test exploits the digestion activity of DNase I added to the sample before extraction to differentiate free naked DNA from the genome encapsidated into virions. Both methods, therefore, identify the presence of infectious virions in blood samples. However, the procedures are laborious and not standardized (8). This pilot study evaluates the clinical utility of a new CMV-RNAemia test to identify active viral replication and guide pre-emptive or prophylaxis strategies in transplant settings, especially in patients receiving LMV.

A total of 254 blood samples from 47 CMV-DNAemia-positive episodes that occurred in 44 transplant recipients (Table 1) were retrospectively tested for the detection and quantification of CMV-RNAemia, using the CMV RNA ELITe MGB kit on ELITe InGenius instrument (ELITechGroup). This is the first available commercial test targeting the virion-associated UL21.5 mRNA, a late transcript highly expressed during lytic infection (10–13). The assay was carried out on plasma samples stored at −80°C until processing. The results of CMV-RNAemia and CMV-DNAemia on the respective whole blood (WB) matrix, collected during virological routine monitoring, are reported in Table 1. Compared to CMV-DNAemia, the CMV-RNAemia showed sensitivity and specificity equal to 32.2% (73/227) and 100% (27/27), respectively; the percentage of agreement between the two methods was 39.3% (100/254, data not shown in Table 1). Specifically, 73 samples resulted positive by both tests (CMV-DNAemia versus CMV-RNAemia), and the correlation in the viral load measurement was high (*r* = 0.755, *P* < 0.001, Table 1). One hundred fifty-four samples were positive only for CMV-DNAemia. Out of these, 57.8% (89/154) were collected during the descending phase of positive CMV-DNAemia episodes (after reaching the peak) or were related to episodes with persistent low CMV-DNAemia levels (below 300 copies/mL). All the latter samples were from patients who were receiving LMV prophylaxis, suggesting that the type of anti-CMV drug administered should be considered during the viral load evaluations. In the analysis, the CMV-DNAemia episodes were stratified into three groups: LMV-prophylaxis, LMV off-label treatment, and pre-emptive therapy. Focusing on cases receiving LMV prophylaxis, CMV-RNAemia resulted positive in 6/25 (24%) episodes (Table 1). Among these, 4/6 (66.7%) were related to clinically significant CMV infections (based on both clinical and laboratory findings), treated by pre-emptive therapy. In 2/4 (50%) cases, genotypic LMV resistance (LMV-R) was documented. Considering the group receiving LMV off-label, six out of seven episodes (85.7%) showed positive CMV-RNAemia (Table 1), and in 3/6 cases (50%), LMV-R was found, leading to switching the treatment to other anti-CMV agents: maribavir, foscarnet, or immunoglobulins. In the remaining three cases, the LMV treatment was continued and combined with a reduced immunosuppressive therapy, until complete viral clearance (two consecutive CMV-DNAemia negative results). In the 12 out of 32 (37.5%) episodes in which CMV-RNAemia was detected during LMV administration, the active viral replication was documented by CMV-viremia and/or DNase tests. These additional methods also confirmed the CMV-RNAemia negative results in the remaining 20/32 (62.5%) episodes, suggesting that the positive CMV-DNAemia in these cases was possibly due to abortive infections. Finally, CMV-RNAemia was positive in all 15 episodes from 14 patients receiving pre-emptive therapy (Table 1).

**TABLE 1:** Characteristics of study population, infective episodes, and samples analyzed

	LMV-prophylaxis	LMV off-label treatment^*b*^	Pre-emptive therapy^*c*^	Total
No. of transplant recipients	23	7	14	44
Male sex	13	5	10	28
Age (mean ± SD)	49 ± 13.2	34 ± 16.1	45 ± 21.2	45 ± 17
Type of transplant				
Hematopoietic stem cells	23	4	6	33
Liver	0	1	3	4
Heart	0	2	2	4
Kidney	0	0	3	3
CMV serostatus D/R^*a*^				
D+/R+	13	0	7	20
D−/R+	10	4	3	17
D+/R−	0	3	4	7
No. of CMV-DNAemia-positive episodes^*d*^	25	7	15	47
CMV-DNAemia-positive/total samples	97/106	35/37	95/111	227/254
Median CMV DNAemia levels in whole blood (copies/mL^*e*^, range)	3.9 × 10^2^ (3 × 10^2^–1.9 × 10^5^)	1.4 × 10^3^ (3 × 10^2^–7.3 × 10^4^)	2.8 × 10^3^ (3 × 10^2^–2.4 × 10^6^)	9.8 × 10^2^ (3 × 10^2^–2.4 × 10^6^)
No. of CMV-RNAemia-positive episodes^*f*^	6	6	15	27
CMV-RNAemia-positive/total samples	14/106	9/37	50/111	73/254
Median CMV-RNAemia levels in plasma (copies/mL, range)	3 × 10 (3 × 10–1.9 × 10^3^)	5.1 × 10 (3 × 10–1.2 × 10^3^)	1.4 × 10^2^ (3 × 10–9.1 × 10^3^)	8.1 × 10 (30–9.1 × 10^3^)
Correlation coefficient between CMV DNAemia and CMV-RNAemia levels	0.571^*g*^	0.237^*g*^	0.759^*h*^	0.755^*h*^
Sensitivity–specificity of CMV-RNAemia vs CMV-DNAemia	14.4%–100%	25.7%–100%	52.6%–100%	32.2%–100%^*i*^

^*a*^ D, donor; R, recipient; +, positive; −, negative.

^*b*^ LMV-off-label due to ganciclovir resistance (three patients receiving solid organ transplant), severe neutropenia (two pediatric hematopoietic stem cell transplant recipients), and positive CMV-DNAemia results when starting LMV (two adult hematopoietic stem cell transplant recipients).

^*c*^ The patients receiving therapy with (val)ganciclovir (*n* = 13) or foscarnet (*n* = 1).

^*d*^ CMV-DNAemia-positive episodes: at least two sequential positive CMV-DNAemia results in whole blood or one more than 300 copies/mL; a mean of 5.4 samples/episodes [±3.5 standard deviation (SD)] was analyzed.

^*e*^ Conversion factor from copies/mL to international units/mL is 0.46.

^*f*^ CMV-RNAemia-positive episodes: episodes with at least one positive result.

^*g*^ Spearman correlation, CMV-DNAemia and CMV RNAemia levels under the lower limit of quantification (300 and 30 copies/ml) were considered equal to 150 and 15 copies/ml, respectively.

^*h*^ Pearson correlation, CMV-DNAemia and CMV RNAemia levels under the lower limit of quantification (300 and 30 copies/ml) were considered equal to 150 and 15 copies/ml, respectively.

^*i*^ The specificity of 100% was also found in 100 additional plasma samples from transplant patients for whom CMV reactivation was ruled out by negative CMV-DNAemia results (data not shown).

**Fig 1:**
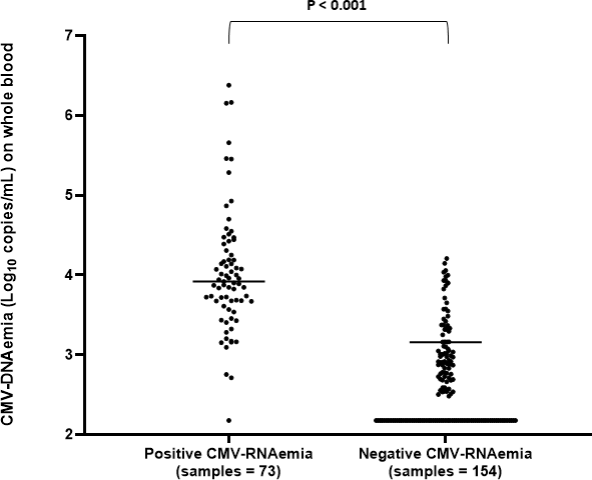
Comparison of CMV-DNAemia levels in samples positive and negative for CMV-RNAemia. Positive values under the lower limit of quantification (300 copies/mL) were reported as equal to 150 copies/mL. Higher median CMV-DNAemia values were observed in specimens positive for CMV-RNAemia than in the negatives: 8,289 copies/mL [interquartile range (IQR): 4,664–21,286.2] vs 373 copies/mL (IQR: 300–1,106.7), respectively; *P* < 0.001 (Mann–Whitney test).

Analyzing all the 227 positive samples (Table 1), the CMV-DNAemia levels were higher in specimens positive for CMV-RNAemia than in those negative (*P* < 0.001, Fig. 1). Considering the three groups of patients, lower median CMV-DNAemia values were found in cases receiving LMV, as prophylaxis or off-label, than in the pre-emptive group (*P* < 0.001, Mann–Whitney test). The results confirmed that detectable low DNAemia levels during LMV administration may reflect abortive rather than productive infection. These data also suggest that CMV-RNAemia could be useful for detecting active CMV replication in patients receiving antiviral therapy, especially with drugs that do not act on the viral DNA polymerase, such as LMV.

**Fig 2:**
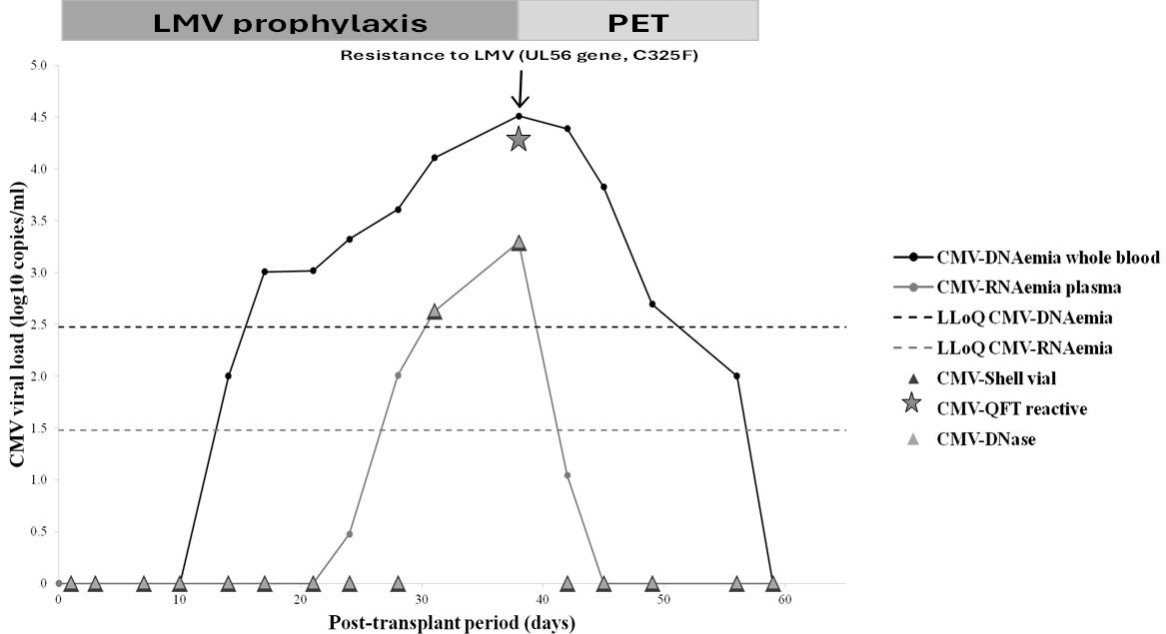
Virological monitoring by CMV-DNAemia and CMV-RNAemia in a hematopoietic stem cell transplant recipient managed by LMV prophylaxis during post-transplant. PET, pre-emptive therapy with foscarnet; LLoQ, lower limit of quantification; CMV-DNase, CMV-DNAemia detection in plasma samples after DNase digestion; CMV-QFT reactive, presence of CMV cell-mediated immune response measured by QuantiFERON-CMV test (QIAGEN). Adult hematopoietic stem cell transplant recipient receiving LMV prophylaxis. An episode of active viral replication was identified by positive results of CMV-RNAemia and of the additional routine methods (CMV-DNase and CMV-viremia). Pre-emptive therapy was started based on both clinical and laboratory findings, obtaining a complete viral clearance as shown by CMV-DNAemia and more rapidly by CMV-RNAemia.

Considering the 27 episodes positive for both markers (Table 1), the CMV-DNAemia and CMV-RNAemia peaks were reached simultaneously, with median levels equal to 11,754 copies/mL (IQR: 7,386.5–26,174) and 81 copies/mL (IQR: positive <30–489.5), respectively. Interestingly, during the descending phase, negative results were obtained earlier with CMV-RNAemia than with CMV-DNAemia (mean time 7 days before, ±6.9 standard deviation), proving more rapidly an efficient viral clearance, as shown in a representative case detailed in Fig. 2. As reported in literature, during this phase, CMV-DNAemia could be detected due to the presence of free viral genome fragments that are released after infected cell degradation and are not necessarily associated with infectious viral particles (3, 4). These observations, together with the previous considerations, show that CMV-DNAemia alone may not be an accurate marker of active CMV replication, especially in patients undergoing LMV.

In this study, CMV-DNAemia was measured in WB samples since previous investigations suggested this blood compartment as a preferable clinical sample for monitoring CMV infection post-transplantation (3, 4, 8). As RNA is an unstable and fragile molecule, plasma samples were immediately frozen at −80°C and analyzed within 3 weeks. At prospective and retrospective testing of CMV-RNAemia in a small group of samples, no significant differences were observed (data not shown), confirming the absence of degradation during storage.

In conclusion, CMV-RNAemia, detected by a standardized user-friendly assay and an automated and integrated system, together with CMV-DNAemia, could provide accurate information on viral load kinetics during post-transplant monitoring of CMV infection, especially in patients receiving LMV. Further studies with larger numbers of samples, including patients undergoing therapy with new anti-CMV drugs such as maribavir, are needed to confirm these preliminary data.
